# Time‐course of clinical symptoms in young people at ultra‐high risk for transition to psychosis

**DOI:** 10.1111/eip.13201

**Published:** 2021-07-22

**Authors:** Anna Meneghelli, Angelo Cocchi, Maria Meliante, Simona Barbera, Lara Malvini, Emiliano Monzani, Antonio Preti, Mauro Percudani

**Affiliations:** ^1^ Programma2000 – Center for Early Detection and Intervention in Psychosis, Department of Mental Health and Addiction Services Niguarda Hospital Milan Italy; ^2^ Department of Mental Health & Drug Abuse, ASST Bergamo Ovest – Treviglio Niguarda Hospital BG Italy

**Keywords:** early intervention, growth mixed model, heterogeneity, psychosis, ultra‐high risk

## Abstract

**Background:**

Ultra‐high risk (UHR) people are a heterogeneous group with variable outcomes. This study aimed at (a) estimating trajectories of response to treatment to identify homogeneous subgroups; (b) establishing the impact on these trajectories of known predictors of outcome in UHR subjects.

**Methods:**

Mixed models of growth curves and latent class growth analysis (LCGA) were applied to the 24‐item brief psychiatric rating scale (BPRS) to measure the response to treatment over 2 years in 125 UHR participants. Group differences were tested on sociodemographic variables and clinical indicators that are known to affect the outcome in UHR people.

**Results:**

BPRS scores decreased across all tested models, with a greater decrease for affective and positive symptoms than for all other dimensions of BPRS. Past admissions to the hospital for psychiatric reasons other than psychosis and the presence of a decline in premorbid functioning before the episode were associated with a slower decrease of BPRS score. LCGA identified three classes, one (82% of participants) with a progressive decrease in the BPRS scores, a second class with a moderate improvement (10%), and a third with no improvement (8%). Those in the ‘no improvement’ class had a higher chance of receiving a diagnosis of psychosis within the spectrum of schizophrenia.

**Conclusion:**

Most UHR individuals that are treated within a specialized service undergo substantial improvement in their psychopathology, but some seem resistant to the protocol of treatment and need close reevaluation within the first 12 months of treatment.

## 1 INTRODUCTION

Within the framework of a transdiagnostic clinical staging model (Shah et al., 2020), increasing attention has been devoted to people showing signs of incipient psychosis, so‐called individuals with ‘ultra‐high risk’ (UHR) for psychosis (Parabiaghi et al., 2019; Yung et al., 2004; Yung & McGorry, 1996). In so far, the transition to psychosis has been the most investigated outcome for UHR samples, with estimates ranging between 18% and 30% depending on follow‐up and sample (Fusar‐Poli et al., 2016; Nelson et al., 2011). However, UHR people are a heterogeneous group (Zhang et al., 2020), with significant comorbid disorders (Albert et al., 2018) that may impact on the course of illness. The exclusive focus on the transition to psychosis may obscure different pathway to remission and recovery in this population (Lin et al., 2015; Simon et al., 2013). Some recent studies attempted to identify separate pathways to symptoms change in UHR samples.

Polari et al. (2018) applied a complex system of stratification of symptoms to a UHR cohort that included 202 individuals, over a 12‐month follow‐up, and identified six trajectories: recovery (35.7%), remission (7.5%), any recurrence (20%), no remission (17.3%), relapse (4.0%) and transition to psychosis (15.8%). Long duration of untreated illness (DUI) and high depression scores were related to the most unfavourable outcomes in this cohort. By applying a more transparent, data‐driven approach based on latent class growth analysis (LCGA) to a larger sample of the same cohort including 304 individuals followed up for an average of 40 months, Hartmann et al., (2020) found two classes with trajectories with mostly parallel slopes (i.e., improving symptoms/functioning over time), which were differentiated mainly by the severity of symptoms and functioning at baseline. In this study, female gender, older age, substance use and lower cognitive functioning were related to the class with the worst outcome. Allswede et al. (2020) applied group‐based multitrajectory modelling, a form of LCGA, to the cohort of the North American Prodrome Longitudinal Study [NAPLS‐2], including 422 individuals that met criteria for a clinical high‐risk (CHR) syndrome per the Structured Interview for Prodromal Risk Syndromes (SIPS; Miller et al., 2003). They found three classes: one of substantial improvement across all investigated domains (30% of the sample), one with moderate impairments across domains and some positive outcome at follow‐up (49%), and one with severe impairments across domains and no positive outcome at follow‐up (22%). Similar patterns of change (i.e., rapid, moderate, or no improvement) were replicated in an independent sample of 133 CHR individuals of the first phase of the NAPLS (NAPLS‐1).

In past studies, LCGA has been used to identify trajectories towards symptoms remission. The main advantage of LCGA is that it is data‐driven, thus allowing the identification of subgroups without the application of superimposed artificial cut‐off points. LCGA also consents to the exploration of the factors that impact class membership. So far, only one study examined the impact of some known predictors of poor outcome in UHR individuals.

### 1.1 Aims

In this study, we applied mixed models of growth curves and LCGA to investigate the response to treatment over 2 years in a sample of UHR patients that were enrolled within an early intervention service. The conditional growth model tends to assume a linear growth trajectory, while the latent class growth mixture model assumes a non‐linear growth. The first model assesses whether a change over time has occurred in the sample and consent to evaluate whether some known predictors influence this change. The second model will allow the identification of subgroups that change in a different way across time, either linearly and non‐linearly. In particular, we wanted (a) estimating trajectories of response to treatment to identify homogeneous subgroups; (b) establishing the impact on these trajectories of known predictors of outcome in UHR subjects.

## 2 METHODS

Data were collected during the routine assessment of the patients participating in the Programma2000, an early intervention service of the Niguarda Hospital of Milan (Cocchi et al., 2008). The study complies with the 1995 Declaration of Helsinki and its revisions (World Medical Association, 2013). Written informed consent was acquired from each participant. The time interval of the study is from 1999 to 2015, when the Programma2000 was reorganized in both the procedures of assessment and the program of cure.

### 2.1 Participants

Programma2000 is a multi‐modal, community‐based outpatient clinic with a served catchment area that includes ~200 000 inhabitants. Inclusion criteria for the UHR diagnosis were: help‐seeking status for distress related to psychosis; aged 17 to 30 years old; to comply with the Personal Assessment and Crisis Evaluation (PACE) Clinic in Melbourne criteria for UHR (Yung et al., 2004; Yung & McGorry, 1996); to have had never received antipsychotic treatment before enrolment; to have had never received a past or present diagnosis of schizophrenia, bipolar disorder or unipolar disorder with psychotic features. People with comorbid medical or neurological disorders or with substance use disorders were excluded and referred to other specialized centres. Recreational substance use not associated with substance use disorder was deemed eligible for treatment and inclusion in the study.

Each patient received a tailored, 3‐years intervention package based on pharmacotherapy, cognitive‐behavioural psychotherapy, psycho‐education, group activities and skills training, family support. Details of this treatment package were reported elsewhere (Cocchi et al., 2008; Meneghelli et al., 2010).

### 2.2 Measures

To assess response to treatment we used the 24‐item brief psychiatric rating scale (BPRS; Overall & Gorham, 1962; Roncone et al., 1999). The BRPS has a Likert scoring in which the listed symptoms were rated from 1 (absent) to 7 (extremely severe). BPRS total scores ranges from 24 to 168, across a gradient of higher levels of psychopathology. The total score of the BPRS is an accepted measure of outcome in clinical trials (Leucht et al., 2007). A judgement of ‘much improved’ rating on the Clinical Global Impression has been equated to a reduction of 58% in the BPRS total score at 1 month (Leucht et al., 2005). We also assessed change over time across the main dimensions of the 24‐item BPRS, as defined by the most reproducible factor structure of this version of the scale (Dazzi et al., 2016). Four invariant subscales are described: Affect (including items on anxiety, guilt, depression, suicidality), Positive Symptoms (hallucinations, unusual thought content, suspiciousness, grandiosity), Negative Symptoms (blunted affect, emotional withdrawal, motor retardation) and Activation (excitement, motor hyperactivity, elevated mood, distractibility) (Dazzi et al., 2016). Details on the changes over time of the scores of these four dimensions of the BPRS are reported in the appendix.

From initial assessment (baseline), the BPRS was administered every 6 months, to assess change over time in levels of psychopathology. Inter‐rater agreement was measured as intra‐class correlation coefficients (ICC), and median values for BPRS, calculated with a two‐way mixed‐effects model, were .78 (95%CI: .71 to .84) in the current sample; these values indicate moderate to good reliability according to current guidelines (Koo & Li, 2016).

Sociodemographic (gender, age at first contact) and clinical indicators that are known to impact outcomes in UHR samples were derived from a detailed interview with the patient and a key informant, usually a parent (see Table 1 for the list of indicators). Details on this procedure were reported elsewhere (Cocchi et al., 2014). In the analysis, continuous variables (age and DUI) were dichotomized to favour comparison with the other categorical variables. Although caution is advised in interpreting the effects of dichotomization of continuous variables (Chen et al., 2007), the statistical analysis is made simpler, leading to an easier presentation of the results.

**TABLE 1:** General characteristics of the sample (*n* = 125)

Gender	
Males	88 (70%)
Females	37 (30%)
Age (years old)	22 (3); range: 16–30
16 to 20 years old	53 (42%)
21 years old or older	72 (59%)
DUI (months)	30 (22); range: 1–60
Less than 12 months	35 (28%)
1 years or more	70 (56%)
The DUI could not be determined	20 (16%)
Past admissions to hospital for psychiatic reasons	
Yes	14 (11%)
No	111 (89%)
History of substance use	
Yes	22 (18%)
No	85 (68%)
Not enough information	18 (14%)
Family history of psychiatric disorders	
Yes	67 (54%)
No	40 (32%)
Not enough information	18 (14%)
Decline in premorbid functioning	
Yes	67 (54%)
No	40 (32%)
Not enough information	18 (14%)
Dropout of treatment after 2 years	
Yes	38 (30%)
No	87 (70%)
BPRS	
Baseline	44 (12); range: 19–99
At 6 months	37 (9); range: 21–76
At 12 months	35 (8); range: 24–76
At 18 months	33 (8); range: 24–62
At 24 months	32 (7); range: 24–59

*Note*: All data are reported as mean (*SD*); range, or counts (percentage).

Conversion to psychosis in the sample was based on the formal DSM‐IV or DSM‐IV‐TR diagnosis of schizophrenia‐spectrum psychosis made by the therapists at the end of the three‐year program.

### 2.3 Statistics

Analyses were carried out with the Statistical Package for Social Sciences (SPSS) version 20 and with dedicated packages running in R (R Core Team, 2018). All tests were two‐tailed (alpha set at *p* < .05). Means with standard deviations or counts and percentages were reported depending on the type of variable (continuous or nominal). Comparisons between groups were by Student's *t* test, ANOVA or Chi‐square (with Yates correction when necessary). When *n* < 5 in some group, we applied Fisher's exact test or the Freeman–Halton extension of Fisher's exact test for contingency tables that are larger than 2 × 2 (Freeman & Halton, 1951). The difference between the score at baseline (minus the minimum score of 24, as in Leucht, 2014) and the score at the end of the 2 years interval of the study (again, minus the minimum score of 24) was calculated to measure improvement on the BPRS across time.

Data were missing for an average of 5% in each time point, with scarce overlap from a point to the other. Overall, less than 20% of data were missing in the sample. We applied multiple imputations by chained equations (mice) method (‘mice’ package version 3.13.0 running in R), to correct for missingness (van Buuren & Groothuis‐Oudshoorn, 2011). We used 20 imputed data sets. Rubin's method was used to derive pool averaging across all imputed data sets.

Changes over time in the variable of interest (BPRS total score) were assessed with a conditional growth model (Rubin, 1996). The model was implemented with the ‘nlme’ package running in R (Pinheiro et al., 2016). The ‘lme’ function was used to implement the models. The impact of sociodemographic variables and some clinical indicators on the response to treatment as measured by the BPRS was also tested. Model fit was investigated according to Nakagawa et al. (2017) with the ‘MuMIn’ package running in R (Barton, 2018). The proportion of variance explained by both fixed (time and group membership) and random factors (intercept and slope at participants' level) was reported as conditional (pseudo)*R* ^*2*^.

LCGA was used to identify separate trajectories of subgroups of patients across time (Jung & Wickrama, 2008). A quadratic function of time at the population level (fixed effects), and a linear function of time at the individual level were used to model changes over time of the BPRS scores. The LCGA models were implemented with the package ‘lcmm’ running in R (Proust‐Lima et al., 2017). The ‘lcmm’ function was used to implement the LCGA models. The best model was expected to minimize the values of the information criteria and to maximize entropy, a measure of the accuracy of participants' classification (0 to 1). The following information criteria were used: the Akaike information criterion (AIC; Akaike, 1987), the Bayesian information criterion (BIC; Schwarz, 1978), the sample‐size adjusted BIC (SABIC, Sclove, 1987). Minimum acceptable entropy values were ≥ .80 (Ramaswamy et al., 1993). In assigning participants to the latent classes, average probabilities per class ≥90% at a minimum were accepted.

Logistic regression in relation to LCGA‐extracted class membership was used to evaluate their links with sociodemographic variables and clinical indicators. Variance explained by the model (0% to 100%) was assessed with the pseudo‐*R* ^2^ McFadden measure (Long, 1997). Since events were rare in some of the classes, we also applied Fisher's exact test to these calculations.

## 3 RESULTS

The study included 125 UHR patients, mostly male participants. The mean age in the sample was 22 years old, with no differences by gender (males: 21.9 ± 3.5 vs. females: 22.2 ± 3.6; t = .69; *df* = 123; *p* = .276). In the sample, 28% of the participants had a DUI less than 12 months (Table 1).

Participants were rarely admitted to the hospital for psychiatric reasons (11%); 54% reported a family history of psychiatric disorders, 13 with non‐affective psychosis among the relatives (20%), and 28 with a relative that received the diagnosis of an affective disorder, either major depressive disorder or bipolar disorder (42%). A minority reported recreational substance use (18%). A decline in premorbid functioning was reported in 54% of participants. Over time, the improvement in symptoms was substantial, with on average 60% decrease of the BPRS scores.

In the sample, 8 UHR patients received a formal diagnosis of schizophrenia‐spectrum psychosis at the end of the 3‐year program.

### 3.1 Impact on symptomatic improvement of sociodemographic and clinical variables

The conditional growth model revealed a substantial decrease of the scores on the BPRS across time, on average of three points every 6 months (Table 2).

**TABLE 2:** Results of the conditional growth models

Variables in the model	Statistics						
	Beta	*SE*	*df*	t	*p*	Log likelihood (LL)	Conditional *R* ^2^
Time	−3.4	.4	498	−7.4	<.0001	LL = ‐2172.4,	61.2%
Gender	−1.4	2.4	123	−.6	.56		
Time x gender	.8	.5	498	1.4	.13	*p* = .0082	
Time	−2.5	.4	498	−6.9	<.0001	LL = ‐2174.4,	57.1%
Age	2.6	2.2	123	1.2	.22		
Time x age	−.8	.5	498	−1.7	.078	*p* = .0156	
Time	−3.7	.4	418	−8.7	<.0001	LL = ‐1809.2,	58.2%
DUI	.05	2.4	103	.02	.98		
Time x DUI	−.5	.5	418	1.0	.30	*p* = .0066	
Time	−2.9	.3	498	−1.8	<.0001	LL = ‐2172.7,	6.6%
Past admissions to hospital	8.3	3.4	123	2.4	.017		
Time x past admissions	−1.2	.8	498	−1.5	.13	*p* = .0007	
Time	−3.3	.3	426	−12.5	<.0001	LL = ‐2322.3,	57.8%
Recreational substance use	−3.6	2.7	105	−1.3	.19		
Time x substance use	.4	.6	426	.7	.47	*p* = .0177	
Time	−3.2	.4	426	−7.6	<.0001	LL = ‐1849.3,	58.2%
Family history	−1.4	2.4	105	−.6	.54		
Time x family history	.2	.5	426	.4	.69	*p* = .0770	
Time	−2.2	.4	426	−5.1	<.0001	LL = ‐1847.9,	58.3%
Premorbid functioning	4.7	2.4	105	1.9	.055		
Time x premorbid functioning	−1.4	.5	426	−2.5	.011	*p* = .0019	
Time	−2.9	.3	498	−9.8	<.0001	LL = ‐2176.4,	57.9%
Dropout	−2.2	2.3	123	−.9	.34		
Time x dropout	.6	.5	498	1.0	.29	*p* = .0472	

A slower decline in BPRS was observed in those with past admissions to the hospital for psychiatric reasons other than psychosis and in those with decline in premorbid functioning before the episode. Age, gender, a family history of psychiatric disorders, a history of recreational substance use, and dropping out of treatment after the first 2 years did not influence the decrease of BPRS scores over time. The fit of all models was good, with Conditional *R* ^2^ above 50%. The effect of the predictor on the explained variance was minimal, with most of the variance attributable to the change over time of the scores on the BPRS.

When the BPRS items were partitioned into four subgroups of affective, positive, negative and activation symptoms, we found that affective (one or more points reduction every 6‐months, *p* < .0001) and positive symptoms (.6 to 1 point reduction every 6‐months, *p* < .0001) were more prone to change than negative symptoms and symptoms of psychomotor activation (.3 or less point reduction every 6‐months, *p* < .05). Thus, changes in total BPRS scores could be attributed principally to changes in affective and positive symptoms.

### 3.2 Trajectories of response to treatment

**FIGURE 1:**
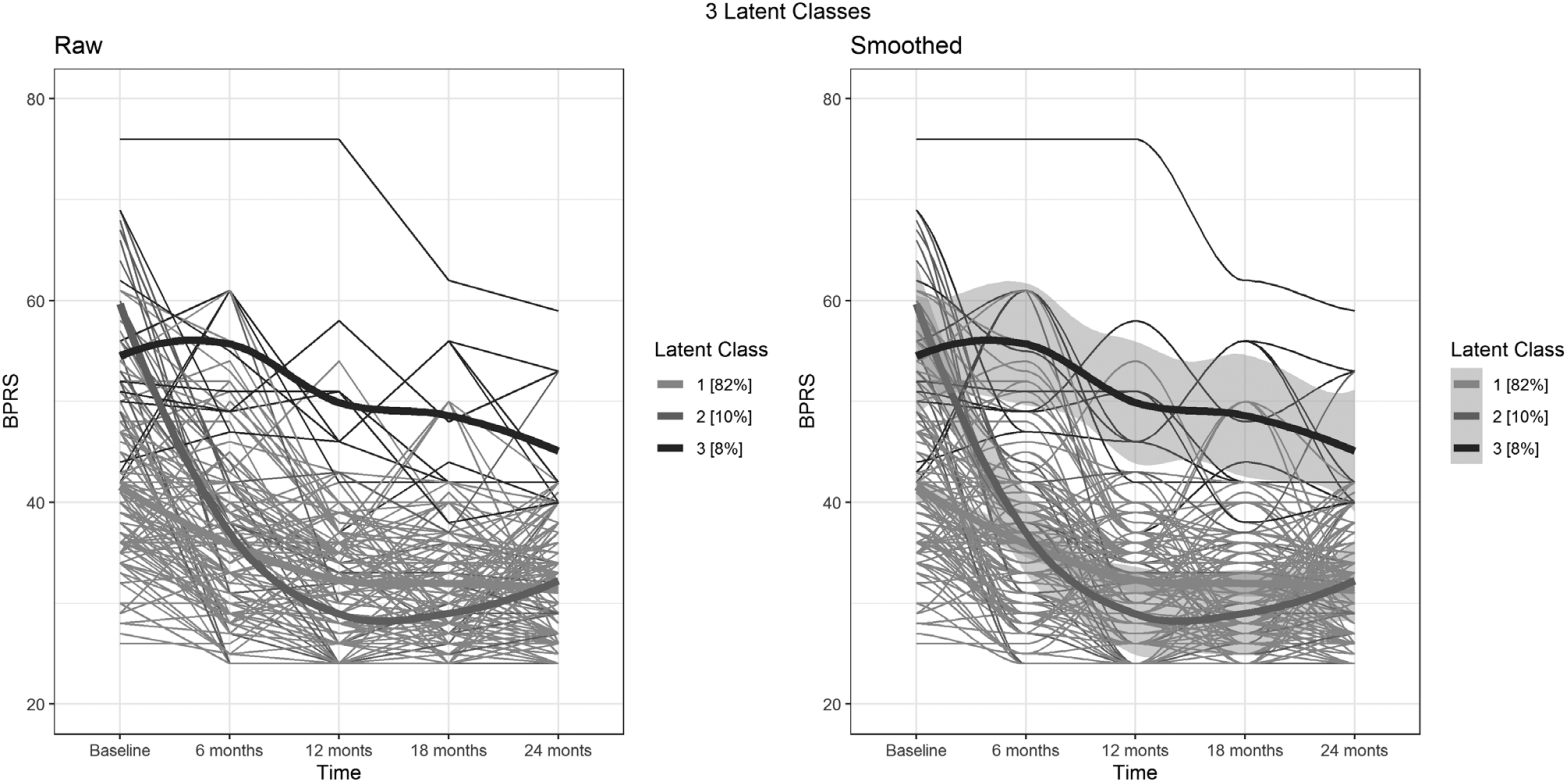
Treatment response trajectories over 2 years. On the left, raw data for all participants (each with their trajectory) and the estimated three‐classes trajectories (wider lines). On the right, the smoothed trajectories of the three classes with the confidence interval, which is tighter in the larger class and larger in those with limited sample size

In the LCGA, the indicators of fit decreased from the 1‐class model to the 6‐class model except for BIC, which increased after the 3‐class model (Table 3). Entropy in the 3‐class model was acceptable (>80%), but the probability of assignment to the class was suboptimal (it was above 90% in just 61% of cases). Both entropy and the maximal probability of assignment to the class increased in the 4‐class, 5‐class and 6‐class models, but in these models, only three classes had participants above 5%, the other retrieved classes had 1% or even less assigned participants. Therefore, the 3‐class model was chosen. Most participants (*n* = 102) were assigned to class 1 (82%); class 2 totalled 13 participants (10%); the remaining participants (n = 10) were assigned to class 3 (8%). Those in class 1 showed a smoothed decrease in BPRS scores starting from values that were, on average, lower than the values observed in the other two classes (Figure 1).

**TABLE 3:** Fit statistics for 1–6 class latent class growth mixture models

n. classes	Log‐likelihood	AIC	BIC	SABIC	Entropy	Posterior probabilities above 90% in each class
1	−2138.05	4290	4309	4287	–	–
2	−2123.01	4268	4299	4264	.91	73%
3	−2098.95	4227	4270	4222	.85	61%
4	−2091.98	4221	4275	4215	.87	74%
5	−2075.90	4197	4262	4190	.87	88%
6	−2070.00	4194	4270	4184	.88	89%

BPRS scores at baseline were 40.9 ± 8.1 in class 1; 62.7 ± 12.8 in class 2; 54.1 ± 11.5 in class 3: F(2;122) = 40.5; *p* < .0001; Tukey's honestly significant difference: *p* < .0001 in the comparison between class 1 and the other classes. Those in class 2 had a sharp decrease in BPRS scores in the first 6 months of treatment and remained in remission in the following months. Those in class 3 did not improve relevantly, although after 24 months of treatment they showed lower BPRS scores than at baseline. Across classes, participants showed some recurrence in symptoms, with worsening (hence, increase in BPRS scores) at 12 or 18 months, with subsequent decrease.

Those in class 3 were more likely to have had a history of past admissions to the hospital for psychiatric reasons. However, the results of the Fisher's exact test did not confirm the association. No other links were found with variables that have the potential to impact the outcome in UHR patients (Table 4).

**TABLE 4:** Baseline clinical variables across the 4 latent classes

	Class 1*	Class 2	Class 3	McFadden *R* ^2^
	*N* = 102	*N* = 13	*N* = 10	
Gender				3.0%
Males	70 (69%)	12 (92%)	6 (60%)	
Females	32 (31%)	1 (8%)	4 (40%)	
OR (95% CI)	1	5.4 (.6–44.0)	.7 (.2–2.6)	
Fisher's exact test		*p* = .105	*p* = .724	
Age (years old)				2.6%
16 to 20 years old	42 (41%)	4 (31%)	7 (70%)	
21 years old or older	60 (59%)	9 (69%)	3 (30%)	
OR (95% CI)	1	.6 (.2–2.2)	3.3 (.8–13.6)	
Fisher's exact test		*p* = .558	*p* = .101	
DUI (months)				.4%
Less than 12 months	29 (33%)	3 (27%)	3 (43%)	
1 years or more	58 (67%)	8 (73%)	4 (57%)	
OR (95% CI)	1	.7 (.2–3.0)	1.5 (.3–7.1)	
Fisher's exact test		*p* = 1.00	*p* = .686	
Past admissions to hospital for psychiatric reasons				3.5%
Yes	8 (8%)	3 (23%)	3 (30%)	
No	94 (92%)	10 (77%)	7 (70%)	
OR (95% CI)	1	3.5 (.8–15.4)	5.0 (1.1–23.3)*	
Fisher's exact test		*p* = .109	*p* = .058	
History of recreational substance use				.2%
Yes	19 (21%)	2 (17%)	1 (17%)	
No	70 (79%)	10 (83%)	5 (83%)	
OR (95% CI)	1	.7 (.1–3.6)	.7 (.1–6.7)	
Fisher's exact test		*p* = 1.00	*p* = 1.00	
Family history of psychiatric disorders				1.2%
Yes	54 (61%)	8 (67%)	5 (83%)	
No	35 (39%)	4 (33%)	1 (17%)	
OR (95% CI)	1	1.3 (.3–4.6)	3.2 (.3–28.9)	
Fisher's exact test		*p* = .762	*p* = .403	
Decline in premorbid functioning				6.4%
Yes	54 (61%)	11 (92%)	2 (33%)	
No	35 (39%)	1 (8%)	4 (67%)	
OR (95% CI)	1	7.1 (.9–57.7)	.3 (.05–1.8)	
Fisher's exact test		*p* = .051	*p* = .224	
Dropout of treatment after 2 years			1.0	.3%
Yes	30 (29%)	5 (38%)	3 (30%)	
No	72 (71%)	8 (62%)	7 (70%)	
OR (95% CI)	1	1.5 (.4–4.9)	2.0 (.2–4.2)	
Fisher's exact test		*p* = .531	3.0 *p* = 1.00	
Latent Class 1 was used as a reference term.				

*Note*: Statistically significant results are in bold. * p<0.05.

At the end of the 3‐year program, those in class 3 were statistically more likely to have received a DSM‐IV/DSM‐IV‐TR diagnosis of schizophrenia‐spectrum psychosis than those in the other two classes: class 1, *n* = 2 (15.4%); class 2, *n* = 3 (2.9%); class 3, *n* = 3 (30%); χ^2^ = 13.08; *df* = 2; *p* = .0014; Freeman–Halton extension of Fisher's exact test: *p* = .0005.

## 4 DISCUSSION

Most UHR individuals who access an early intervention service undergo improvement and progressive change of their symptoms. The peak improvement occurs after 12 months of treatment. However, a subgroup of those who improve may undergo cycles of worsening and further improvement across time. UHR people that have higher baseline scores of psychopathology have different trajectories than those observed in the majority of those who improve. A subgroup undergoes a rapid amelioration followed by a smooth worsening of their symptoms. Another subgroup does not improve relevantly over the first 2 years of treatment, and this group has a greater risk of transition to psychosis. Most change in symptoms was attributable to a decrease of affective and positive symptoms. Other dimensions of the BPRS, such as negative symptoms, were less prone to changes.

Gender and age did not emerge as predictors of symptoms trajectories as they were in the study of Hartmann et al., (2020), and this may depend on a shorter range of age in our sample and a greater prevalence of males than in the study of Hartmann et al., (2020). The lack of impact of substance use is coherent with past investigation on the topic (Farris et al., 2020; Hartmann et al., 2020). All other known predictors of outcome in UHR samples were not related to the trajectories that have been identified in this study, except a decline in premorbid functioning before the episode, which was related to the reduced improvement of psychopathology as measured by the BPRS. The level of functioning was related to the trajectories of change that were identified by LCGA in the study of Hartmann et al., (2020) but not in the study of Allswede et al. (2020). However, the results of this study are more similar to the findings of Allswede et al. (2020), who identified three trajectories of change (rapid, moderate and no improvement), than to those of Hartmann et al., (2020), who found two trajectories with progressive and constant improvement. Heterogeneity of the samples may partially explain these differences, as well as the different measures to which the LCGA was applied.

This study is not exempt from limitations, and albeit it was based on state‐of‐the‐art statistics it lacked a control group. This was the main limitation of the study and depended on the lack of an agreement with the psychiatric services operating in neighbour areas about the application of an assessment detailed and repeated as the one that it is implemented in the Programma2000. The sample size was too limited to detect specific associations of the longitudinal classes with some of the predictors of outcome that were investigated in the study. Moreover, the use of a 6‐month interval for repeated assessment might have prevented a better definition of the trajectories. The availability of only binary data (yes or not) for some predictors might have limited the identification of relevant relationships. Finally, because of sample size, we were unable to apply a method to approximate the distribution of trajectories across time on multiple variables as in the NAPLS‐2 study (Allswede et al., 2020). Thus we were unable to determine whether some specific dimension of symptoms (whether affective, positive, negative, or activation) contributed most to the diversion of classes 2 and 3 from the pattern of class 1, which was the majority in the sample.

## 5 CONCLUSIONS

Most UHR individuals undergo substantial improvement when treated within an early intervention service. However, some of these patients seem resistant to the multimodal protocol of treatment that is administered and probably need close reevaluation within the first 12 months of treatment to identify factors that are related to scarce improvement.

## CONFLICT OF INTEREST

The authors declare that they have no conflict of interest.

## Data Availability

Research data are not shared since informed consent only allowed analysis and publication of collected data as a summary or group description.

## Appendix A.

Mean, *SD* and range of values for the four dimensions of the BPRS across time, from baseline assessment to 24 months. Data are reported in succession for Affective symptoms, positive symptoms, negative symptoms and activation

**Table:**

BPRS	Time	Affective symptoms	Positive symptoms	Negative symptoms	Activation
	Baseline	14 (4); range: 5–32	10 (4); range: 6–26	6 (3); range: 3–18	8 (3); range: 6–29
	At 6 months	11 (4); range: 5–23	8 (2); range: 6–21	5 (3); range: 3–17	7 (2); range: 6–18
	At 12 months	9 (3); range: 5–21	7 (2); range: 6–21	5 (3); range: 3–17	7 (2); range: 6–17
	At 18 months	9 (3); range: 5–19	7 (2); range: 6–17	4 (2); range: 3–10	7 (2); range: 6–15
	At 24 months	9 (3); range: 5–19	7 (2); range: 6–16	4 (2); range: 3–16	7 (2); range: 6–15
